# Supplementary material for: Calibration of in-frame indel variant effect predictors for clinical variant classification
Source: bioRxiv. 2026 Apr 18:2026.04.15.718599. Preprint. [Version 1] doi: 10.64898/2026.04.15.718599 (PMC13105015; doi:10.64898/2026.04.15.718599)
Supplement: Supplement 1 [file media-1.pdf]

Supplementary Information

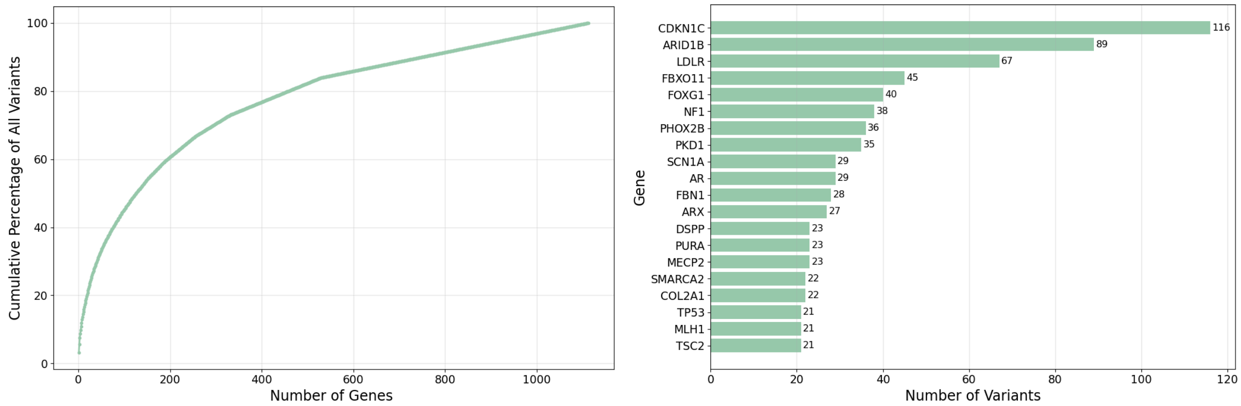

Supplementary Figure 1. Distribution of ClinVar 2023 short in-frame indel variants across disease-associated genes. (left) Cumulative percentage of ClinVar 2023 variants accounted for by the top N genes ranked by variant count. (right) Top 20 genes represented in ClinVar 2023 dataset.

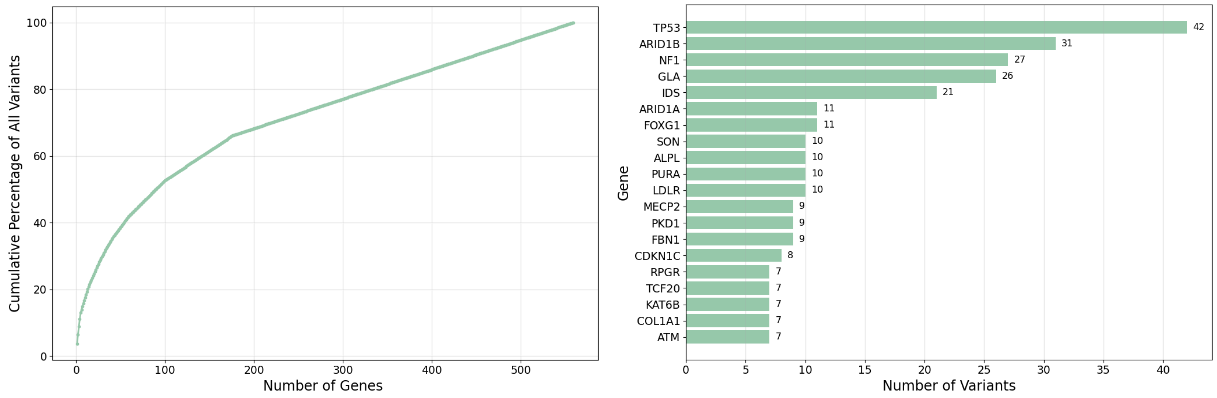

Supplementary Figure 2. Distribution of ClinVar 2025 short in-frame indel variants across disease-associated genes. (left) Cumulative percentage of ClinVar 2025 variants accounted for by the top N genes ranked by variant count. (right) Top 20 genes represented in ClinVar 2025 dataset.

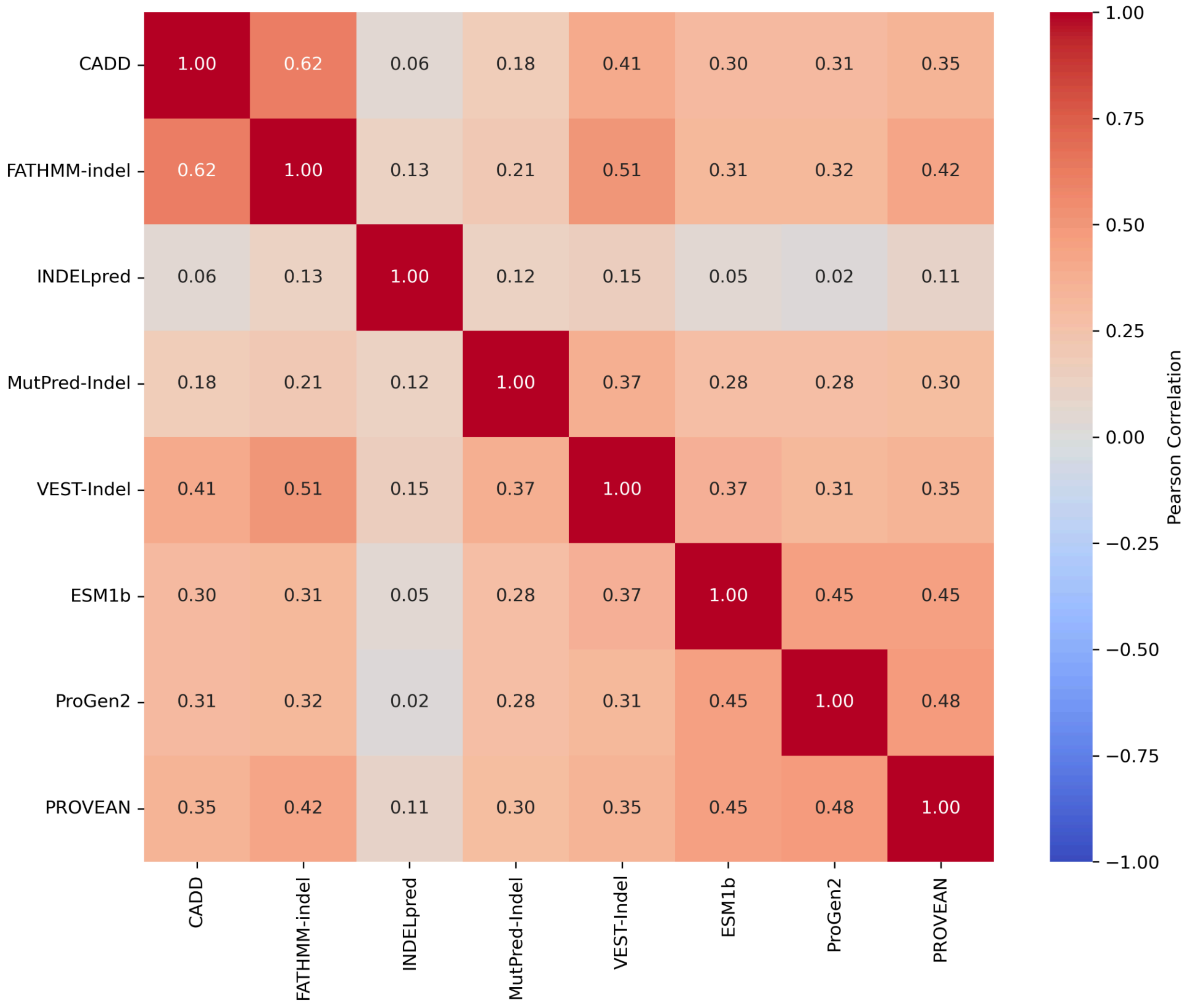

Supplementary Figure 3. Pairwise correlation for all methods on gnomAD data set. We calculated the Pearson correlation coefficient for each pair of tools on their intersection of scored variants. Scores were flipped for tools that consider lower scores to be pathogenic (ESM1b, ProGen2, and PROVEAN). We observe notably lower levels of correlation compared to missense variant effect predictors (Pejaver et al., 2022).

	Variants after removing training data	Variants scored
CADD	3625	3622
FATHMM-indel	3593	3472
INDELpred	1783	1688
MutPred-Indel	3017	3017
VEST-Indel	2780	2668
ESM1b	3625	3579
ProGen2	3625	3579
PROVEAN	3625	3576

Supplementary Table 1. Tools used for analysis. Number of ClinVar 2023 variants provided to each tool for scoring after removing training data and the final number of variants scored for each.

Deletions

	-4	-3	-2	-1	Indet.	+1	+2	+3	+4
CADD		33	197	273	1860	251			
FATHMM-indel	287	138	194	156	575	388	774		
INDELpred	148	9	44	31	941	58			
MutPred-Indel			291	109	1067	343	146	188	
VEST-Indel		276	247	38	409	206	303	389	
ESM1b					1505	487	310	278	
ProGen2				550	851	323	610	246	
PROVEAN	426	41	240	194	291	979	95	311	

Insertions

	-4	-3	-2	-1	Indet.	+1	+2	+3	+4
CADD				136	698	144	30		
FATHMM-indel				112	597	251			
INDELpred	47	0	79	17	314				
MutPred-Indel				228	473	121	51		
VEST-Indel				253	337	60	108	42	
ESM1b					755	79	165		
ProGen2					756	116	127		
PROVEAN			71	314	316	36	262		

Supplementary Figure 4. ClinVar 2023 set variant counts for deletions (top) and insertions (bottom) that met evidence strength level for each tool.

Deletions

	-4	-3	-2	-1	Indet.	+1	+2	+3	+4
CADD		5	40	68	674	105			
FATHMM-indel	41	25	57	37	186	136	375		
INDELpred	42	3	21	10	714	50			
MutPred-Indel			66	28	289	155	71	139	
VEST-Indel		56	55	13	198	76	109	194	
ESM1b					409	206	125	140	
ProGen2				115	265	110	250	140	
PROVEAN	66	14	62	65	83	372	44	173	

Insertions

	-4	-3	-2	-1	Indet.	+1	+2	+3	+4
CADD				20	160	40	19		
FATHMM-indel				9	124	82			
INDELpred	4	1	16	5	187				
MutPred-Indel				36	110	42	24		
VEST-Indel				44	71	19	41	14	
ESM1b					151	28	52		
ProGen2					148	26	57		
PROVEAN			9	66	59	11	89		

Supplementary Figure 5. ClinVar 2025 set variant counts for deletions (top) and insertions (bottom) that met evidence strength level for each tool.

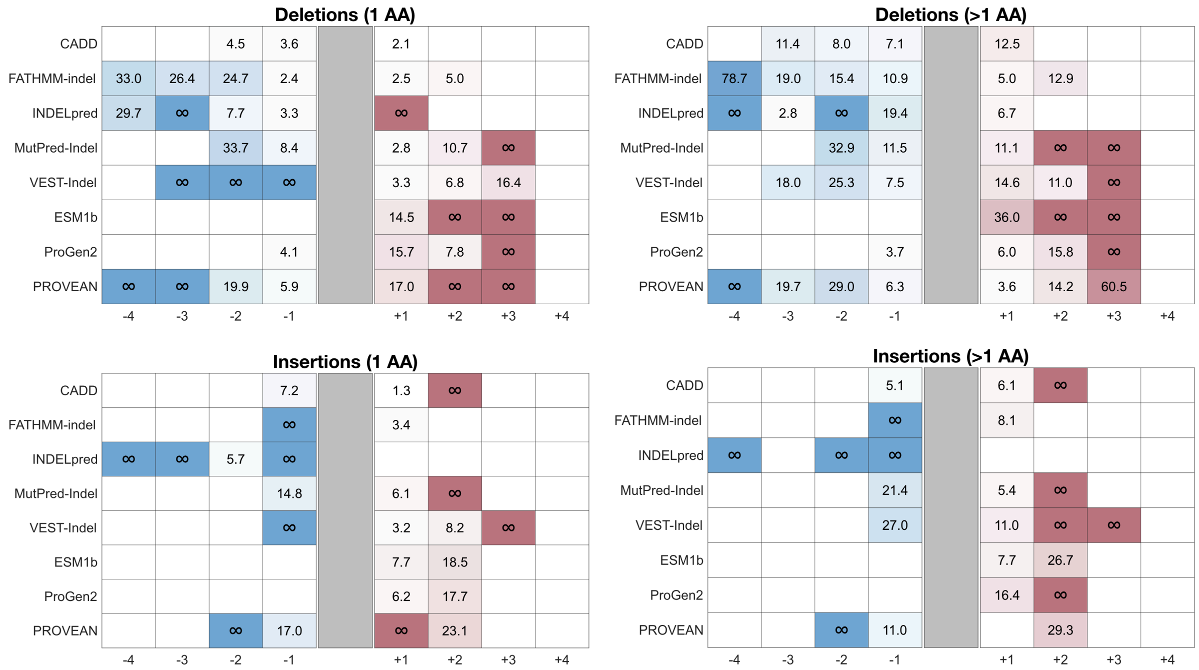

Supplementary Figure 6. Likelihood ratios calculated on ClinVar 2025 set calculated separately for shorter (one amino acid change) and longer (greater than one amino acid change and shorter than 17 amino acid change) deletions and insertions. The specific grouping was made to roughly split the available variants in half. The expected likelihood ratios for deletions are 2.39 (supporting, +1), 5.69 (moderate, +2), 13.59 (moderate, +3) and 32.42 (strong, +4). The expected likelihood ratios for insertions are 3.25 (supporting, +1), 10.55 (moderate, +2), 34.28 (moderate, +3) and 111.34 (strong, +4).

	Deletions		Insertions	
	1 amino acid	> 1 amino acid	1 amino acid	> 1 amino acid
CADD	1288	1326	347	661
FATHMM	1240	1272	325	635
INDELpred	556	675	154	303
MutPred-Indel	1012	1132	298	575
VEST-Indel	834	1034	256	544
ESM1b	1274	1306	341	658
ProGen2	1274	1306	341	658
PROVEAN	1275	1302	343	656

Supplementary Table 2. Counts of scored ClinVar 2023 variants per tool for one amino acid indels versus longer indels. The specific grouping into short versus long in-frame indels was made to roughly split the available variants in half.

	All genes	Disease-associated genes
	Scored (total 4548 variants)	Scored (total 1140 variants)
CADD	4547	1140
FATHMM-indel	4533	1136
INDELpred	4541	1134
MutPred-Indel	4220	1052
VEST-Indel	4442	1133
ESM1b	4223	1053
ProGen2	4223	1053
PROVEAN	4314	1106

Supplementary Table 3. Number of rare in-frame indels scored for all genes and disease-associated genes from RGP probands.

Additional information about indel computational predictors

Web servers:
CADD: https://cadd.gs.washington.edu/score
VEST-Indel: https://cravat.us/CRAVAT/
PROVEAN: http://provean.jcvi.org/genome_submit_2.php?species=human (accessed via Safari web browser)
FATHMM-indel: http://indels.biocompute.org.uk/
MutPred-Indel: https://mutpred.mutdb.org/mutpredindel/

CADD and VEST-Indel scores are freely available for non-commercial use; commercial uses require a license. FATHMM-indel is freely available via the web server at the link above. MutPred-Indel allows free use of the web site for non-commercial and commercial use, with the number of variants per query limited to 100. MutPred-Indel source code and software are freely available for non-commercial use and require a license for commercial use. The PROVEAN web server was accessed at the link above without registration using the Safari web browser; the server was operational at the time of analysis despite being listed as retired by JCVI. The standalone PROVEAN software remains available for download under the GNU General Public License v3. INDELpred, ESM1b, ProGen2, and MutPred-Indel were run locally using code downloaded from their respective GitHub repositories or website for MutPred-Indel. ESM1b is released under the MIT License and ProGen2 under the BSD-3-Clause License. INDELpred does not specify an explicit code license.
